# High-Precision, Self-Powered Current Online Monitoring System Based on TMR Sensors Array for Distribution Networks

**DOI:** 10.3390/s25051473

**Published:** 2025-02-27

**Authors:** Zhengang An, Lei Zhang, Zhi Wang, Yanyun Fan, Zhiwei Zu, Zhengzhe Li, Dachao Li

**Affiliations:** State Key Laboratory of Precision Measurement Technology and Instruments, Tianjin University, Tianjin 300072, China; anzhengang@tju.edu.cn (Z.A.); 2022202200@tju.edu.cn (Z.W.); fanyanyun0614@tju.edu.cn (Y.F.); zuzhiwei@tju.edu.cn (Z.Z.); 1024202065@tju.edu.cn (Z.L.)

**Keywords:** current sensor, TMR sensor, current online monitoring system, power distribution network, self-powered

## Abstract

Establishing a maintenance-free current sensing network across the entire power grid to facilitate wide-area online monitoring is crucial for realizing a smart grid. However, distribution networks (DNs) frequently lack effective real-time current monitoring owing to the complexity of load types, extensive line distribution, and numerous branches. In this study, we propose a high-precision, self-powered online current monitoring system that integrates a TMR sensors array module, a main control module, a current transformer (CT) power harvesting module, and current online monitoring software. The TMR sensors array module boasts a measurement range of 0–300 A and a high sensitivity of 25.38 mV/A. To address wire eccentricity errors in array sensors, we develop a neural network-based correction algorithm, which identifies wire positions and applies correction coefficients, achieving high accuracy with an average error of 1.23%. Current data are wirelessly transmitted to software terminals via 4G communication for remote monitoring. Furthermore, the CT power harvesting module converts magnetic energy from the power grid into electrical energy, ensuring that the system is self-powered. Validation through continuous 24-h monitoring of DNs demonstrates the system’s high precision and stability. This work presents an effective solution for high-accuracy online current monitoring in DNs.

## 1. Introduction

Smart grids increasingly incorporate distributed generation systems powered by renewable sources such as solar, wind, and hydro power [1,2,3]. However, the intermittent and volatile nature of these energy sources introduces challenges such as frequency fluctuations, voltage flickers, and harmonics, adversely affecting the power quality in distribution networks (DNs) [4,5,6,7,8]. Additionally, the centralized integration of distributed loads, exemplified by electric vehicle charging stations, further intensifies the load pressure on DNs, increasing the risk of grid faults and power outages [9,10]. As a result, high-precision current measurement has become a critical requirement for ensuring the safe and stable operation of DNs [11,12,13,14].

Recent advancements in current sensing technology for DNs have been noteworthy. For example, Li et al. [15] proposed a current monitoring system based on a composite integrator Rogowski coil for transient current measurement in DNs. By optimizing the structure of the Rogowski coil probe, the system achieved a MHz-level measurement bandwidth with an error controlled within 5%. Sanchez et al. [16] designed a TMR current sensor based on a Wheatstone bridge structure. By optimizing the PCB layout and incorporating a temperature compensation circuit, the sensor achieved a ±30 A DC measurement range with a high sensitivity of 9.8 mV/A. Under various temperature testing conditions, the sensor demonstrated a sensitivity temperature coefficient as low as 0.031%/°C. TMR sensors have garnered significant attention due to their high stability, sensitivity, resolution, low-temperature drift, and low power consumption [17]. However, several challenges remain before TMR-based current online monitoring technology can be practically applied to DNs. First, sensors in DNs often face numerous environmental interference factors and installation-induced eccentricity, which severely impact measurement accuracy. Second, the extensive distribution and numerous branches of DNs pose significant challenges for the deployment and maintenance of monitoring devices, highlighting the urgent need for stable self-powered modules tailored for power distribution sensors [18]. Finally, with the advancement of digital and smart grids, there is a pressing need to develop system-level wireless monitoring terminals and online monitoring platforms specifically designed for DNs [19,20,21,22,23]. Against this backdrop, developing a current monitoring system with high precision, a wide measurement range, self-powered capability, and wireless transmission is crucial for achieving real-time monitoring in DNs and ensuring grid safety [24,25,26,27,28,29,30].

In this study, we propose a high-precision, self-powered online current monitoring system tailored to address the challenges of accurate real-time current monitoring in DNs. The system integrates a TMR sensors array module, a main control module, a CT power harvesting module, and current online monitoring software. The TMR sensors array module boasts a measurement range of 0–300 A and a high sensitivity of 25.38 mV/A. To mitigate wire eccentricity errors, we develop a neural network-based correction algorithm that identifies wire positions and incorporates position-specific correction factors, achieving high precision with an average error of 1.23%. Current data are wirelessly transmitted via 4G communication to the software terminal for remote monitoring. Furthermore, the CT power harvesting module converts magnetic energy from the power grid into electrical energy, enabling self-powering. Our 24-h continuous monitoring simulation in DNs demonstrates the system’s high accuracy and stability, providing an effective solution for precise online current monitoring in DNs.

## 2. Results and Discussion

### 2.1. Current Monitoring System Configuration

**Figure 1:**
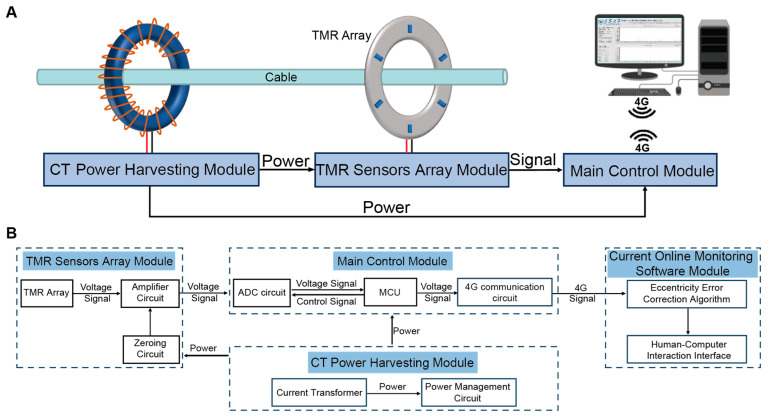
Current online monitoring system based on TMR sensors array. (**A**) Schematic diagram of the current monitoring system configuration. (**B**) Workflow diagram of the current monitoring system.

Figure 1 shows the schematic design of the current monitoring system for DNs, which mainly consists of the TMR sensors array module, the CT power harvesting module, the main control module, and the current online monitoring software module.

The 6-TMR circular sensors array of the TMR sensors array module converts the magnetic field generated by the cable current into a voltage signal, which is then amplified and zeroed before being transmitted to the main control module. The main control module includes an MCU, an analog-to-digital conversion (ADC) circuit, and a 4G communication circuit. The MCU controls the ADC circuit to synchronously collect the signals from the TMR sensors array and transmits the digital signals to the current online monitoring software via 4G communication. The current online monitoring software, installed on a remote terminal (such as a computer), is equipped with data processing and analysis algorithms, including current inversion and eccentricity error correction algorithms, to calculate the current values and correct the eccentricity errors. The CT power harvesting module converts the magnetic energy in the DNs cable into electrical energy using the CT. Then, through the power management circuit, the electrical energy is rectified, filtered, and regulated to supply the required operating voltage for each module, achieving self-powered operation of the system.

### 2.2. Design and Characterization of TMR Sensors Array Module

The current ranges widely in DNs. For example, the current ranges from a few amperes to several tens of amperes in a single-phase 220 V system, while up to hundreds of amperes in a 380 V system [31]. Therefore, the target measurement range of the current monitoring system is 300 A. According to the Biot-Savart Law, the maximum magnetic field strength generated by a cable carrying 300 A is 29 Gs. In this study, the high-precision TMR2103 is selected as the core sensing chip for the system. The TMR2103 features a linear operating range of 0~40 Gs and a sensitivity of 6.0 mV/V/Gs. Testing of the input–output characteristics (Figure S1) of the TMR2103 indicates that its output voltage exhibits a standard linear relationship with the magnetic field strength.

**Figure 2:**
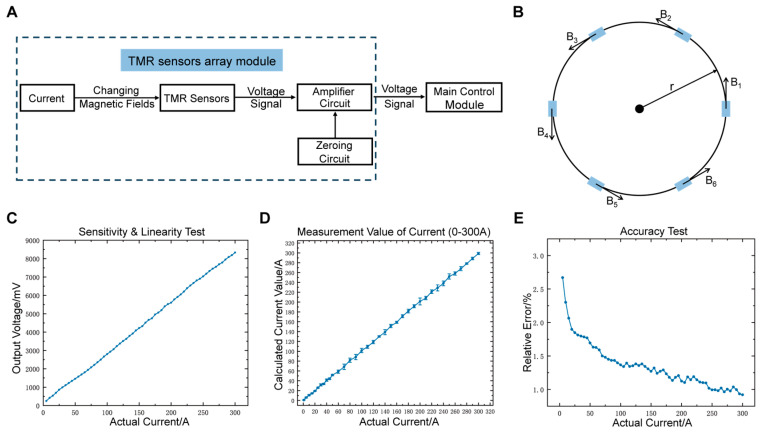
Design and characterization of TMR sensors array module. (**A**) Block diagram of the TMR sensors array module. (**B**) Structure of the TMR sensors array. (**C**) Relationship between the voltage output of the sensing module and the measured current. (**D**) Relationship between the sensing module’s measured current and the actual current. (**E**) Variation of the sensing module’s relative current error with the measured current.

Figure 2A shows the block diagram of the TMR sensors array module, which consists of a 6-TMR sensors array, an amplifier circuit, and a zeroing circuit. Figure 2B shows the structure of the TMR sensors array. The wire is vertically placed at the center of the array, with six TMR sensor chips evenly distributed around it, with a radius of 20 mm. The principle for calculating the current using the TMR sensors array is based on the current inversion algorithm. Each TMR chip measures the magnetic field component in the tangential direction of the circumference. The magnetic field generated by an infinitely long straight current-carrying conductor at the *i*-th TMR sensor chip is given by:

(1) $$B_{i}=\frac{\mu_{0}I}{2\pi r}$$

where $\mu_{0}$ is the vacuum permeability (4π × 10^−7^ H/m), *r* is the array radius (in meters), and *I* is the measured current (in amperes). The output voltage of the *i*-th TMR chip is:

(2) $$V_{i}=K_{i}\cdot B_{i}$$

where $K_{i}$ is the sensitivity coefficient of the *i*-th TMR chip. The conductor current *I* can be determined from the sum of the output voltages of *N* TMR chips as follows:

(3) $$I=\frac{{2\pi r\cdot \sum_{i=1}^{N}\;V}_{i}}{\mu_{0}\sum_{i=1}^{N}\;K_{i}}$$

The array-based measurement method enhances resistance to interference from non-uniform magnetic fields and reduces the impact of conductor misalignment, thereby improving measurement accuracy. The voltage signals from the TMR array are amplified before output. To mitigate the influence of environmental magnetic fields, a zeroing circuit based on a voltage follower is designed. The circuit allows for offset adjustment of the TMR array output by rotating a potentiometer knob.

The performance of the sensing module is tested. Figure 2C shows the relationship between the output voltage and the measured current (0~300 A), with the fitted equation V=25.38I−8.5 mV, yielding a sensitivity of 25.38 mV/A and a nonlinearity of 0.396%. This indicates that the output voltage is almost ideally linear, accurately reflecting small current variations. Figure 2D compares the measured current values with the actual values in the 0~300 A range, showing excellent agreement and maintaining a good linear relationship in a wide current range. In the accuracy test (Figure 2E), the relative error gradually decreases from 10 A to 300 A, stabilizing below 1.5% for currents above 150 A, with an average error of 1.23% and a standard deviation of 0.32%. These results demonstrate the excellent accuracy performance of the sensing module.

### 2.3. Eccentricity Error Correction Algorithm Based on Neural Network

**Figure 3:**
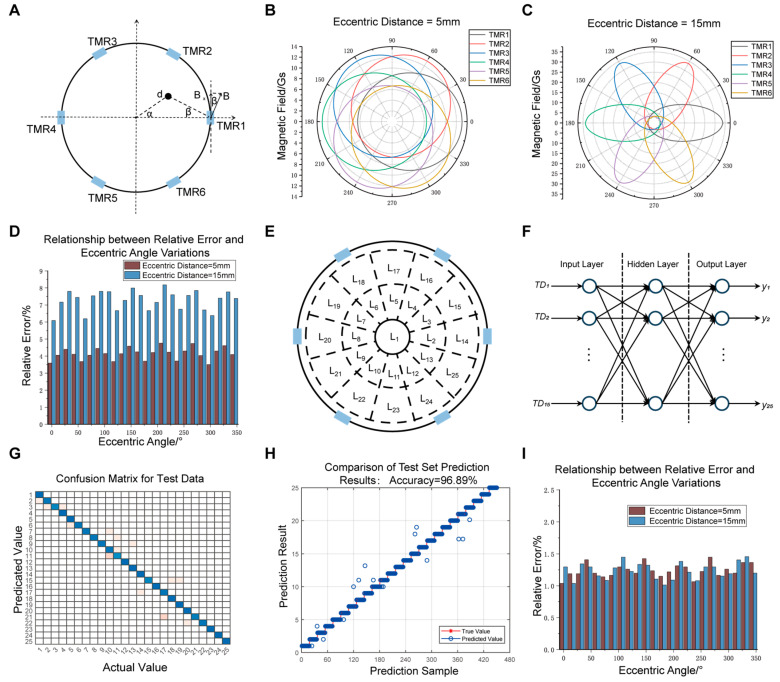
Eccentricity error correction algorithm based on neural network. (**A**) Schematic diagram of wire eccentricity. (**B**) Variation of magnetic field intensity at six TMR chips with respect to the eccentric angle when the eccentric distance is 5 mm. (**C**) Variation of magnetic field intensity at six TMR chips with respect to the eccentric angle when the eccentric distance is 15 mm. (**D**) System eccentric error before correction. (**E**) Positional partitioning of the wire within the array. (**F**) Schematic diagram of neural network structure. (**G**) Confusion matrix for the test dataset. The blue diagonal regions represent the number of correctly classified samples for each conductor eccentric position, while yellow areas indicate the presence of misclassified samples, with darker shades representing a higher number. (**H**) Prediction results of the test dataset. (**I**) System eccentric error after correction.

During operation of the array-based sensor, eccentricity issues arise due to environmental changes or installation errors [32,33], as shown in Figure 3A. Eccentricity affects the magnetic field distribution within the array, leading to significant measurement errors. Figure 3B,C show the variations in TMR chips’ magnetic field strength with respect to the eccentric angle under different eccentric distances. As the eccentric distance increases, magnetic field fluctuations become larger and vary periodically with the eccentric angle. The eccentricity test results (Figure 3D) show that at a current of 50 A, the relative error can reach up to 8% under different degrees of eccentricity.

To address this, an eccentricity error correction algorithm based on neural networks is designed. The conductor’s position within the circular array is divided into 25 regions (Figure 3E). The neural network identifies the wire’s position and constructs a corresponding correction factor for each position, which is then incorporated into the current inversion algorithm to achieve error correction. Using the TMR chips’ output voltages and their difference (including 15 pairs such as TMR1, TMR1-TMR2, TMR1-TMR4, etc.) as inputs and the conductor positions (L1 to L25) as outputs, a three-layer BP neural network is constructed, as shown in Figure 3F. Figure 3G,H show the confusion matrix and recognition results of the test data. The points on the diagonal indicate correctly classified samples, where the model’s output matches the actual position. In contrast, the points off the diagonal represent misclassified samples, with their vertical coordinates indicating the incorrectly predicted conductor positions. Only a few positions had recognition errors, with an overall recognition accuracy of 96.89%.

After identifying the wire position, the eccentricity correction coefficient of the TMR chips is obtained as follows: The magnetic field strength of the wire at each TMR sensor’s sensitive axis direction at the identified position is calculated, then compared with the magnetic field strength generated when the wire is at the center of the array. When the wire is eccentric, as shown in Figure 3A, with position $\left(x_{c},y_{c}\right)$, eccentric angle α, eccentric distance *d*, and the radius of the TMR array is *r*, the distance from the conductor to the *i*-th TMR chip is:

(4) $$r_{i}=\sqrt{{\left(x_{i}-x_{c}\right)}^{2}+{\left(y_{i}-y_{c}\right)}^{2}}$$

Thus, the magnetic field $B_{i}$ at the *i*-th TMR chip is:

(5) $$B_{i}=\frac{\mu_{0}I}{2\pi r_{i}}$$

The angle between $B_{i}$ and the TMR chip’s sensitive axis is β, so the component of $B_{i}$ along the TMR sensitive axis is $B_{i}\cos\beta$. When the conductor is at the center of the array, the magnetic field at each TMR chip’s sensitive axis is $B=\frac{\mu_{0}I}{2\pi r}$. Therefore, the correction factor $\gamma_{i}$ for each TMR chip at eccentric position can be determined as:

(6) $$\gamma_{i}=\frac{B}{B_{i}\cos\beta }=\frac{\sqrt{{\left(x_{i}-x_{c}\right)}^{2}+{\left(y_{i}-y_{c}\right)}^{2}}\cos\beta }{r}$$

Based on this analysis, Table S1 shows the TMR chip correction factors at the 25 positions. The correction factors are then multiplied by the sensitivity coefficients $K_{i}$ in the current inversion algorithm to obtain new sensitivity coefficients $K_{i}^{\prime }$:

(7) $$K_{i}^{\prime }=\gamma_{i}\cdot {\;K}_{i}$$

Thus, the corrected current $I^{\prime }$ is:

(8) $$I^{\prime }=\frac{{2\pi r\cdot \sum_{i=1}^{N}\;V}_{i}}{\mu_{0}\sum_{i=1}^{N}\;K_{i}^{\prime }}=\frac{{2\pi r\cdot \sum_{i=1}^{N}\;V}_{i}}{\mu_{0}\sum_{i=1}^{N}\;\gamma_{i}K_{i}}$$

We perform eccentricity testing after applying the eccentricity error correction. Figure 3I shows the relationship between relative error and eccentric angle for a test current of 50 A. The relative error decreases from 3.5–7% to 1.0–1.4% via correction. By introducing the neural network correction model, the monitoring system achieves a significant improvement in measurement accuracy under different eccentric positions, particularly remaining stable under higher current conditions.

### 2.4. Design and Characterization of CT Power Harvesting Module

To achieve a self-powered system, we first analyze the power consumption requirements of each module, as shown in Table 1. The power consumption of the TMR sensors array module is 313.2 mW, which of the main control module is 474.5 mW, and the total power consumption is 787.7 mW.

**Figure 4:**
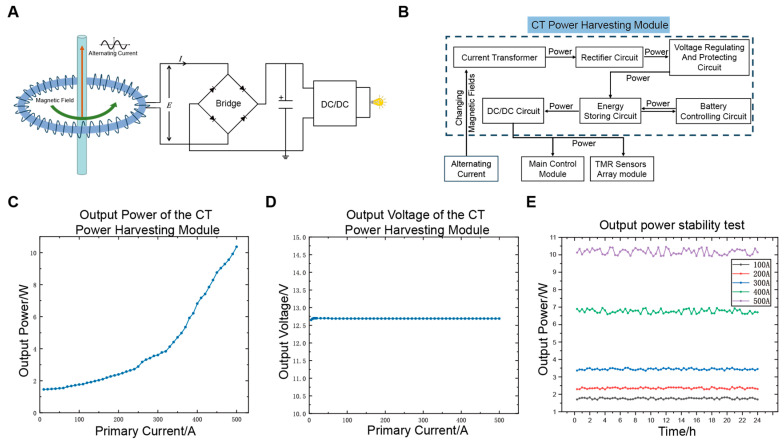
Design and characterization of CT power harvesting module. (**A**) Schematic diagram of the CT power harvesting module principle. (**B**) Block diagram of the CT power harvesting module composition. (**C**) Variation of the output power of the CT power harvesting module with the measured current. (**D**) Variation of the output voltage of the CT power harvesting module with the measured current. (**E**) Output power stability test of the CT power harvesting module under different measured currents.

CT energy harvesting, with its high energy conversion efficiency, simple structure, and easy installation, offers significant advantages in energy harvesting for transmission lines. We develop a CT-based power harvesting module, with its principle and block diagram shown in Figure 4A,B. The CT power harvesting module consists of a CT, rectification circuit, energy storage circuit, voltage conversion circuit, over-voltage protection circuit, and battery control circuit. The CT is placed around the cable to harvest electrical energy through electromagnetic induction. The rectification circuit converts the AC output from the CT into DC. The energy is then stored in the energy storage circuit to stabilize the power output and meet the system’s transient power demands. The voltage conversion circuit converts the high voltage output from the energy storage circuit into the working voltage required by each module. The over-voltage protection circuit prevents voltage fluctuations caused by short circuits in the DNs. Additionally, the battery control circuit activates a backup battery when energy harvesting is insufficient, ensuring that the system continues to operate steadily under low current conditions.

We test the output voltage and power of the CT power harvesting module. Figure 4C shows the relationship between the output power and the measured current. The output power increases with the current, with a minimum value of 1.5 W, which meets the system’s power consumption requirements. Figure 4D displays the curve of output voltage versus the measured current. The output voltage remains stable at approximately 12.7 V, demonstrating high voltage stability. Figure 4E presents the 24-h output power stability test, where the module maintains a high level of output power stability.

### 2.5. Application in Current Online Monitoring of Distribution Networks

**Figure 5:**
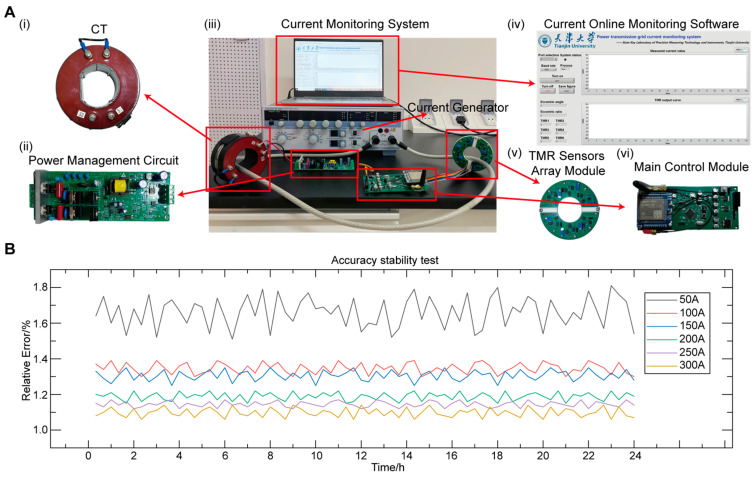
Configuration of system performance testing platform and system accuracy and stability testing. (**A**) Current monitoring system performance testing platform. (**i**) Current transformer. (**ii**) Power management circuit. (**iii**) Physical diagram of the system composition. (**iv**) Upper computer interface. (**v**) Physical diagram of the sensing module. (**vi**) Physical diagram of the main control module. (**B**) System accuracy and stability test diagram.

To evaluate the accuracy and stability of the system, we build an experimental platform as shown in Figure 5A to validate the current monitoring system. During a continuous 24-h test, the system operates normally, Figure 5B shows the results of sampling and processing current data. Within the 300 A current measurement range, the system’s accuracy remains within 1.8%, with nonlinearity within 1.4% in the 100 A to 300 A range, demonstrating excellent long-term stability and accuracy.

## 3. Conclusions

In conclusion, we propose a high-precision, self-powered current online monitoring system to achieve accurate online monitoring of DNs. The developed TMR sensors array module achieves a high sensitivity of 25.38 mV/A within a current range of up to 300 A, with a nonlinear error of less than 0.4%. To minimize eccentricity error in the array-based sensor, we design a neural network-based eccentricity error correction model, achieving a position recognition accuracy of 96.89% and high accuracy with an average error of 1.23%. Additionally, the current data are wirelessly transmitted to the software terminal via 4G communication for remote online monitoring. Moreover, a power harvesting module based on CT is designed to convert magnetic energy from the power grid into electrical energy, enabling self-powered. The system is developed and tested as a laboratory prototype, reaching TRL 6, and demonstrates high accuracy and stability in a 24-h continuous monitoring simulation of currents in DNs.

However, some limitations remain. The neural network-based correction model reduces eccentricity errors, but its accuracy may be affected by external magnetic interference in DNs. Additionally, the prototype has only been validated in a controlled environment, and its long-term reliability under fluctuating grid loads and environmental conditions remains unverified. To address these challenges, future work will focus on implementing adaptive filtering or multi-sensor fusion to enhance robustness. Extensive field testing will be conducted to assess performance, ensuring feasibility for large-scale deployment in DNs.

## 4. Experiment

### 4.1. Fabrication of the Current Monitoring System

The current online monitoring system based on the TMR sensors array for DNs consists of four main components: the TMR sensors array module, the main control module, the CT power harvesting module, and the current online monitoring software module. The detailed fabrication process for each module is as follows:

**TMR sensors array module.** The TMR sensors array module consists of a TMR sensors array, amplifier circuit, and zeroing circuit. The TMR sensors array consists of six TMR chips (TMR2103 MTD, Shenzhen, China) evenly distributed in a circular arrangement with a radius of 20 mm. The amplifier circuit is a first-order amplifier based on the operational amplifier (AD8421, TI, Dallas, TX, USA). The zeroing circuit uses a voltage follower based on the operational amplifier (AD847, Analog Devices, Norwood, MA, USA). These circuits were integrated into two semicircular PCBs, forming a split-ring structure (40 mm inner diameter, 60 mm outer diameter), which allows the module to be mounted or detached easily onto cables.

**Main control module.** The main control module consists of an MCU (STM32F103RCT6, STMicroelectronics, Geneva, Switzerland), an ADC (Analog Devices AD7606, Analog Devices, Norwood, MA, USA) circuit, and a 4G (EC200, Quectel, Shanghai, China) communication circuit. These circuits were integrated into a dual-layer PCB with dimensions of 60 mm × 120 mm. The STM32 controlled the AD7606, which had an analog input voltage range of ±10 V and a sampling rate of 50 kHz. The output pins of the TMR sensors array module were connected to the input pins of the AD7606. The 4G communication circuit interfaced with the STM32 via a UART serial port, and a high-speed USB interface was included as a backup.

**CT power harvesting module.** The CT power harvesting module consists of a CT (LDK3-10, 300 turns, LDK, Shenzhen, China), rectification circuit, energy storage circuit, over-voltage protection circuit, voltage conversion circuit, and battery control circuit. The rectification circuit is a bridge rectifier made of four diodes (1N4001, ON Semiconductor, Phoenix, AZ, USA). The energy storage circuit uses a 30 F supercapacitor. The over-voltage protection circuit consists of two 16 V 5 W Zener diodes connected in reverse parallel to form a transient voltage suppression branch. The voltage conversion circuit utilizes multiple voltage regulators: LM2596-ADJ (high voltage to 12 V, Texas Instruments, Dallas, TX, USA), A1212S (12V to ±12V, Analog Devices, Norwood, MA, USA), LM7809 (12 V to 9 V, STMicroelectronics, Geneva, Switzerland), LM7909 (−12 V to −9 V, STMicroelectronics, Geneva, Switzerland), LM7805 (12 V to 5 V, STMicroelectronics, Geneva, Switzerland) and AMS1117 (5 V to 3.3 V, Advanced Monolithic Systems, San Jose, CA, USA). These circuits were integrated into a dual-layer PCB with dimensions of 55 mm × 155 mm.

### 4.2. Characterization of the Current Monitoring System

We set up a test platform (Figure 5A) to test the performance of the TMR current monitoring system. The test platform consists of a high-precision AC constant current source (2558A, Yokogawa Electric Corporation, Tokyo, Japan), a programmable AC constant current source (ATC70000, Anmtake Electronics, Shenzhen, China), and the current monitoring system. The YOKOGAWA 2558A generates AC currents ranging from 0.3 mA to 60 A with an accuracy of ±0.06%, while the Anmtake ATC70000 provides a wider range of 0 to 1 kA with an accuracy of ±0.3%. The high precision of both devices ensures their suitability as standard reference current sources.

**Characterization of the TMR sensing chip.** To test the input–output performance of the TMR2103, we set up a test platform consisting of an FB523 magnetoresistance experiment instrument (Jingke Instrument, Shenzhen, China), a Gauss meter, and Helmholtz coils. The experiment instrument provided a current of 0 to 1 A to the Helmholtz coils, generating a uniform magnetic field in the center of the coil. The Gauss meter was used to measure the magnetic field strength in this region. The TMR2103 chip was fixed at the center of the two coils to measure the magnetic field generated by the Helmholtz coils. The experiment instrument collected and displayed the output voltage of the TMR2103. The chip was powered at 1 to 7 V, and the coil current was increased from 0 to 1 A. The magnetic field strength at the measurement points and the output voltage of the chip were recorded, obtaining the input–output characteristics of the TMR2103.

**Characterization of the TMR sensors array module.** To test the sensitivity and linearity of the sensing module, an Anmtake ATC70000 was used to generate a 50 Hz sinusoidal AC current ranging from 0 to 300 A. The sensing module was vertically mounted on the cable, with the cable positioned at the center of the TMR array. The current was linearly increased from 0 A to 300 A with a step size of 5 A. The output voltage of the sensing module was recorded to obtain the sensitivity and linearity characteristics of the sensing module. To assess the accuracy of the sensing module, the six TMR voltage signals were input into the current inversion algorithm in MATLAB (R2020a). The calculated current values were compared with the actual current values, and the relative error was computed to generate the accuracy testing curve for the sensing module. To evaluate the impact of conductor eccentricity on the sensing module accuracy, YOKOGAWA 2558A generated high-precision currents ranging from 10 A to 50 A, and different eccentric distances were created using a 3D printed fixture, which was used with the sensing module to position the cable at a fixed eccentric location within the array. The relative error was then calculated in the same manner as described earlier.

**Characterization of the CT power harvesting module.** To test the output voltage of the CT power harvesting module, the CT was mounted on the cable, and an Anmtake ATC70000 was used to generate a 50 Hz sinusoidal AC current ranging from 0 to 500 A. The current linearly increases from 0 A to 500 A with a step size of 10 A. A multimeter was used to measure the output voltage of the power harvesting circuit. To test the output power, a high-power sliding rheostat was connected to the output of the power harvesting circuit. A multimeter was used to measure the voltage across the sliding rheostat. The resistance of the rheostat was gradually decreased from its maximum value. When the voltage reading on the multimeter began to drop, the voltage across the rheostat, denoted as *U*, was recorded. Then, the connection between the rheostat and the energy harvesting circuit was disconnected, and the resistance value *R* of the rheostat was measured using the multimeter in resistance mode. The output power of the CT power harvesting module was then calculated using the formula $W=\frac{U^{2}}{R}$.

### 4.3. Eccentricity Error Correction Algorithm Configuration

We collected 60,000 current data samples from 25 different conductor positions, with 500 × 6 groups of data for each position, to construct a three-layer BP neural network. The inputs of the neural network consist of the outputs of six TMR chips and the differences between adjacent and opposite chips, yielding a total of 15 input features, specifically: *V_TMR1_*, *V_TMR2_*, *V_TMR3_*, *V_TMR4_*, *V_TMR5_*, *V_TMR6_*, *V_TMR1_-V_TMR2_*, *V_TMR2_-V_TMR3_*, *V_TMR3_-V_TMR4_*, *V_TMR4_-V_TMR5_*, *V_TMR5_-V_TMR6_*, *V_TMR6_-V_TMR1_*, *V_TMR1_-V_TMR4_*, *V_TMR2_-V_TMR5_*, *V_TMR3_-V_TMR6_*. The network outputs correspond to the 25 conductor positions, with 500 samples per position. Of these, 350 samples (70%) were used for training, and 150 samples (30%) were reserved for testing. In MATLAB, the neural network was configured with 15 input nodes, 30 hidden layer nodes, and 25 output nodes. The mapminmax function was used to normalize the input data, and the network was trained using the train function with a maximum of 1000 iterations and a target training error of 10^−10^. The identified conductor position index (1 to 25) from the neural network was then applied to the current inversion algorithm. Based on Table S1, the switch function was utilized to select the TMR chips’ correction coefficients at the identified position to calibrate the current values.

### 4.4. Application in Current Online Monitoring of Distribution Networks

We used the experimental platform shown in Figure 5A to validate the current monitoring system for distribution network applications. The sensing system underwent continuous 24-h operation testing to evaluate its accuracy and stability. An Anmtake ATC70000 was used to generate 50~300 A currents to simulate distribution network currents. The cable was fixed at the center of the TMR sensors array module, and current measurement was taken continuously for 24 h. The data was sent to the current online monitoring software, and MATLAB was used for periodic sampling and calculation of the data. The computed current values were then sent to LABVIEW (2020) for real-time display.

## Figures and Tables

**Table 1:** Power consumption analysis of the current monitoring system.

	TMR Sensors Array Module			Main Control Module		
Composition	TMR Array	Amplifier Circuit	Zeroing Circuit	ADC Circuit	MCU	4G Communication Circuit
Power consumption	198 mW	86.4 mW	28.8 mW	60 mW	49.5 mW	365 mW
Total	787.7 mW					

## Data Availability

Data are contained within the article and Supplementary Materials.

## Supplementary Materials

The following supporting information can be downloaded at: https://www.mdpi.com/article/10.3390/s25051473/s1, Figure S1: Output characteristics of the TMR2103 were tested under supply voltages ranging from 1 V to 7 V; Figure S2: TMR output performance test platform; Figure S3: (A) The diagrams of the fixed wire at different eccentric positions; (B) Wire fixture and the TMR sensors array module fixture; Figure S4: Test procedure for the output power of the CT power harvesting module: TMR output performance test platform; Figure S5: Circuit schematics of the TMR sensors array module; Figure S6: Circuit schematics of the main control module; Figure S7: Circuit schematics of the CT power harvesting module; Table S1: The TMR chip correction factors at the 25 conductor’s positions; Table S2: Performance parameters of the utilized current sources in this work.
