# Supplementary material for: High-Precision, Self-Powered Current Online Monitoring System Based on TMR Sensors Array for Distribution Networks
Source: Sensors (Basel). 2025 Feb 27;25(5):1473. doi: 10.3390/s25051473 (PMC11902725; doi:10.3390/s25051473)
Supplement: Supplementary file 1 [file sensors-25-01473-s001.zip › sensors-3459915-supplementary.pdf]

Supporting Information

# High-Precision, Self-Powered Current Online Monitoring System based on TMR sensors array for Distribution Networks

Zhengang An[#], Lei Zhang[#,*], Zhi Wang, Yanyun Fan, Zhiwei Zu, Zhengzhe Li, Dachao Li[*]

State Key Laboratory of Precision Measurement Technology and Instruments, Tianjin University, Tianjin, 300072, China.

[#]Zhengang An and Lei Zhang contributed equally to this work.

[*]Corresponding author
*E-mail*: zhangleitd@tju.edu.cn (Prof. Zhang Lei), dchli@tju.edu.cn (Prof. Li Dachao)

**Keywords:** current sensor; TMR sensor; current online monitoring system; power distribution network; self-powered

**Content of the SupplementaryInformation**

**Figure S1**:  Output characteristics of the TMR2103 were tested under supply voltages ranging from 1V to 7V.

**Figure S2** TMR output performance test platform.

**Figure S3** (A) The diagrams of the fixed wire at different eccentric positions; (B) Wire fixture and the TMR sensors array module fixture.

**Figure S4 Test procedure for the output power of the CT power harvesting module.** (i) Anmtake ATC70000 is used to generate AC current; (ii) Reduce the resistance of the sliding rheostat; (iii) The voltage reading on the multimeter begins to decrease; (iv) Measure the resistance R of the sliding rheostat.

**Figure S5** Circuit schematics of the TMR sensors array module.

**Figure S6** Circuit schematics of the main control module.

**Figure S7** Circuit schematics of the CT power harvesting module.

**Table S1**. The TMR chip correction factors at the 25 conductor's positions.

**Table S2**. Performance parameters of the utilized current sources in this work.

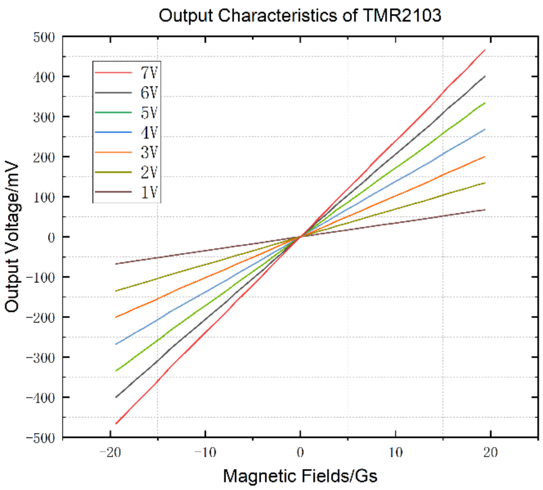


**Figure S1** Output characteristics of the TMR2103 were tested under supply voltages ranging from 1V to 7V.

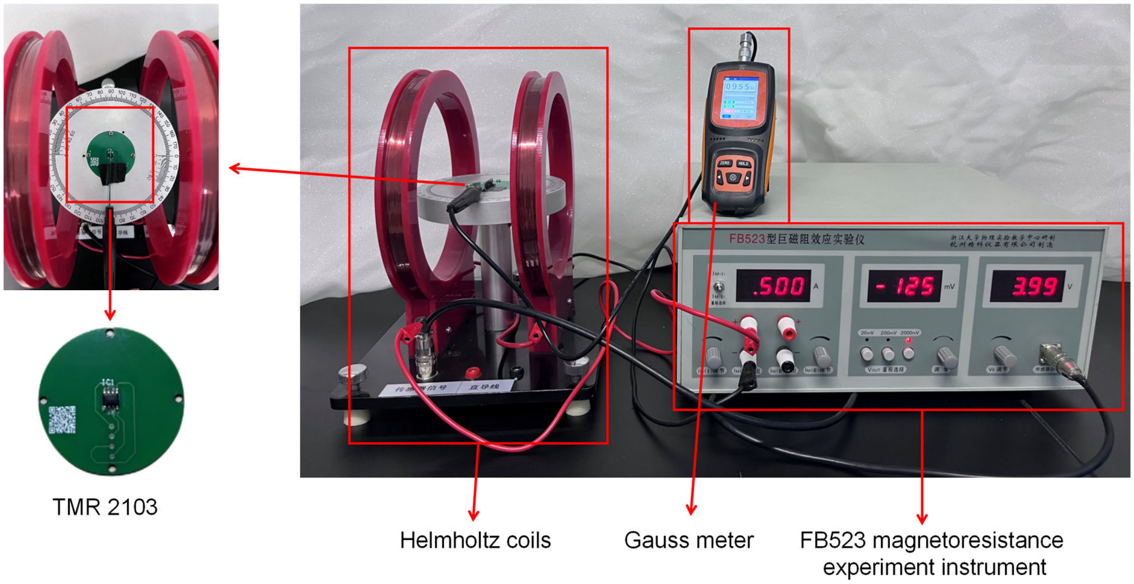


**Figure S2** TMR output performance test platform.

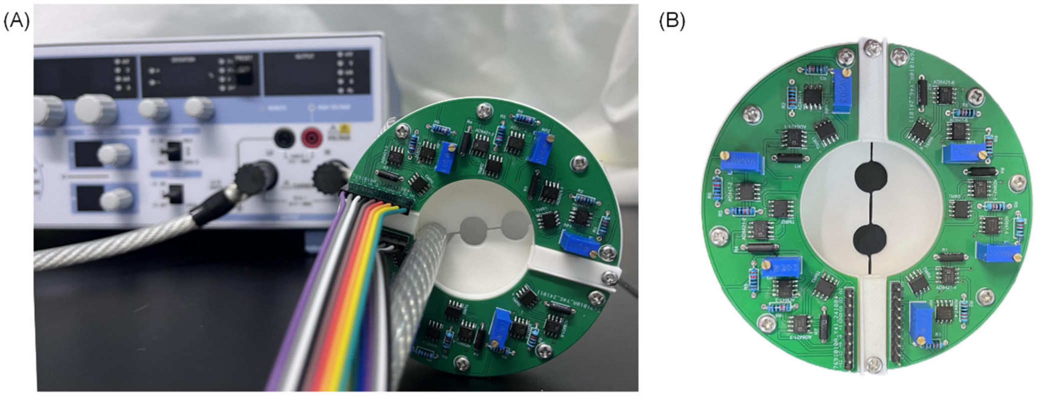


**Figure S3** (A) The diagrams of the fixed wire at different eccentric positions; (B) Wire fixture and the TMR sensors array module fixture.

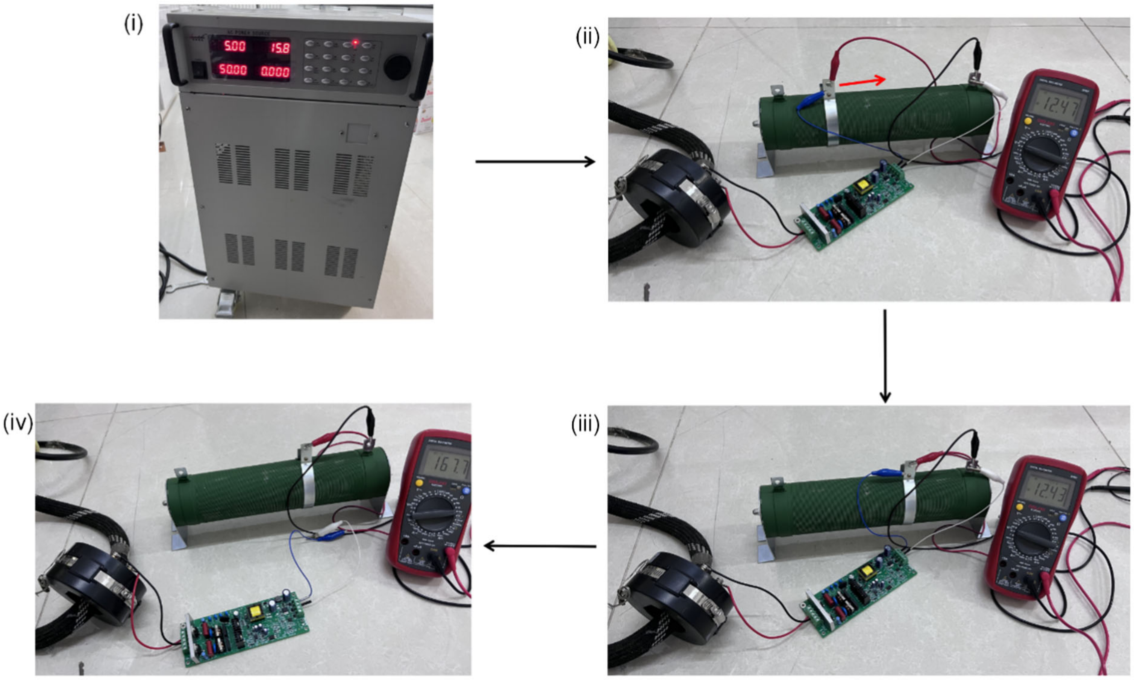


**Figure S4 Test procedure for the output power of the CT power harvesting module.** (i) Anmtake ATC70000 is used to generate AC current; (ii) Reduce the resistance of the sliding rheostat; (iii) The voltage reading on the multimeter begins to decrease; (iv) Measure the resistance R of the sliding rheostat.

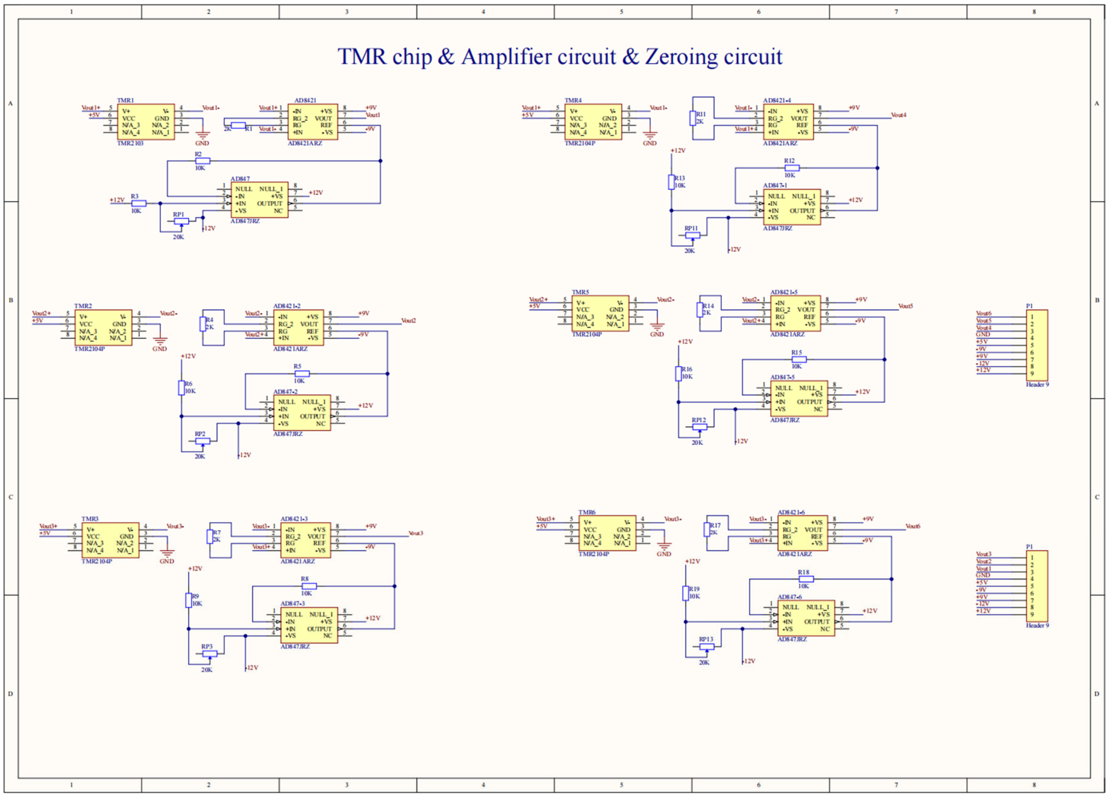


**Figure S5** Circuit schematics of the TMR sensors array module.

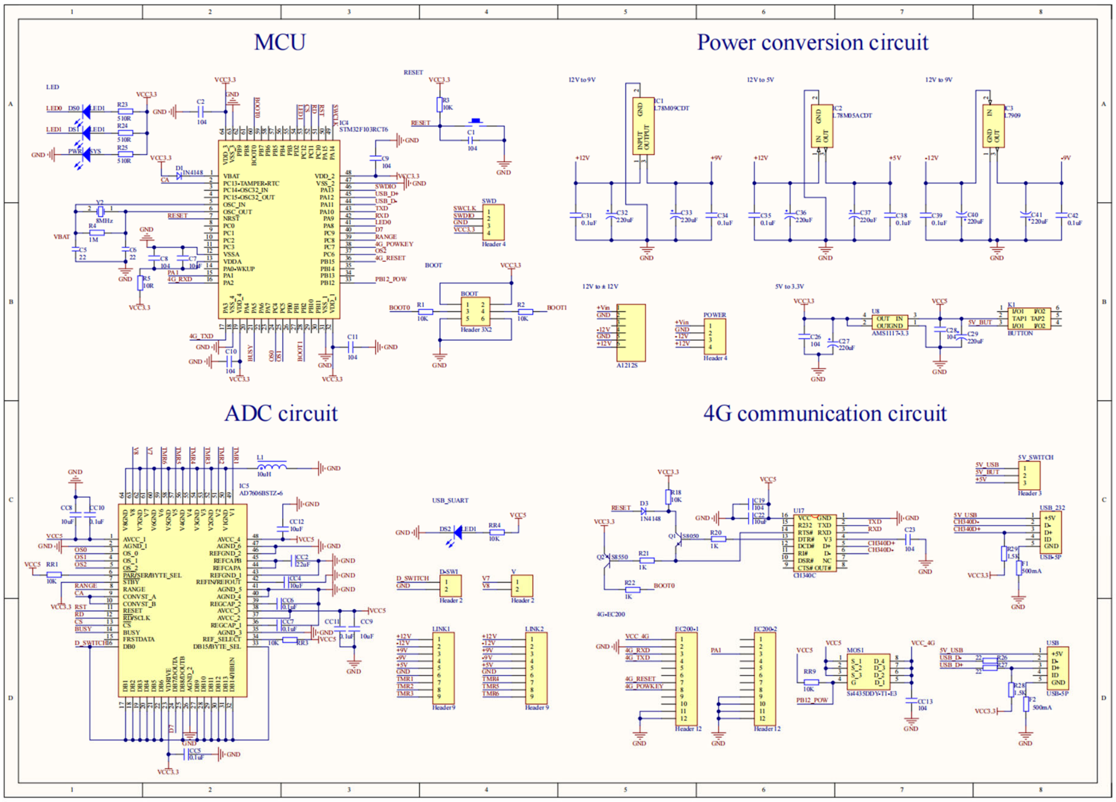


**Figure S6** Circuit schematics of the main control module.

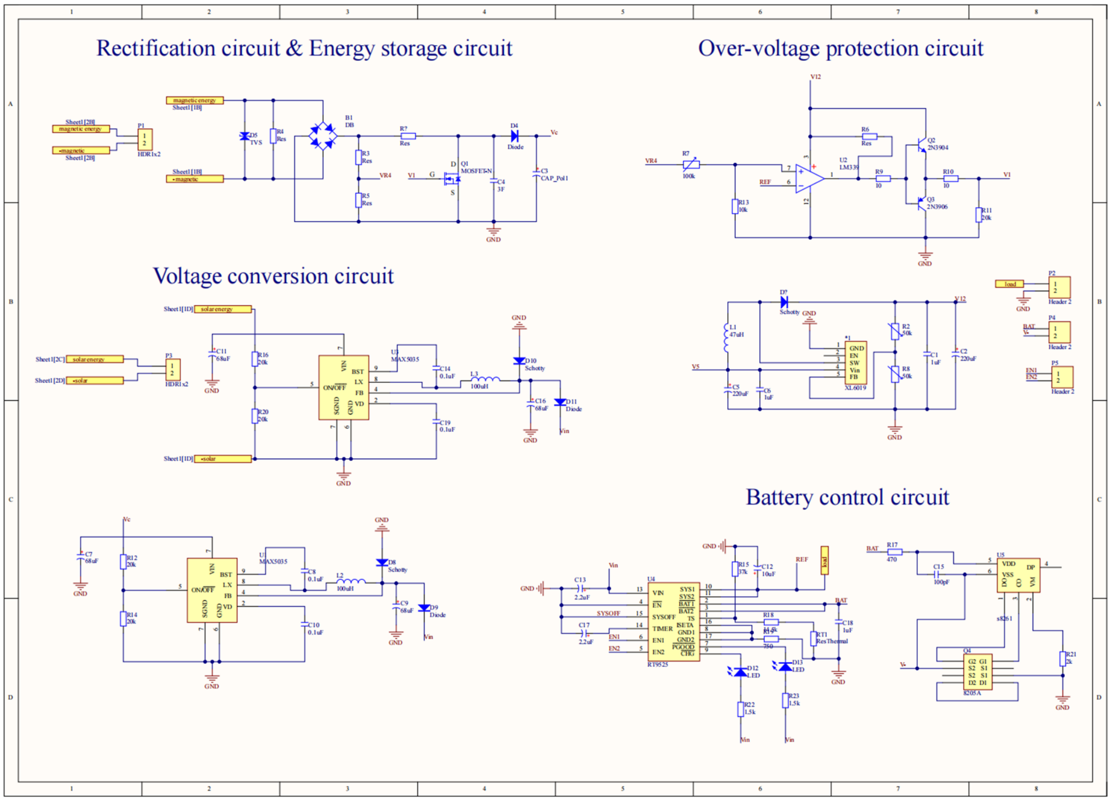


**Figure S7** Circuit schematics of the CT power harvesting module.

**Table S1**. The TMR chip correction factors at the 25 conductor's positions.

| | TMR1 | TMR2 | TMR3 | TMR4 | TMR5 | TMR6 |
|---|---|---|---|---|---|---|
| $L_1$ | 1 | 1 | 1 | 1 | 1 | 1 |
| $L_2$ | 0.77 | 0.93 | 1.15 | 1.23 | 1.15 | 0.93 |
| $L_3$ | 0.85 | 0.85 | 1.04 | 1.19 | 1.19 | 1.04 |
| $L_4$ | 0.93 | 0.77 | 0.93 | 1.15 | 1.23 | 1.15 |
| $L_5$ | 1.04 | 0.85 | 0.85 | 1.04 | 1.19 | 1.19 |
| $L_6$ | 1.15 | 0.93 | 0.77 | 0.93 | 1.15 | 1.23 |
| $L_7$ | 1.19 | 1.04 | 0.85 | 0.85 | 1.04 | 1.19 |
| $L_8$ | 1.23 | 1.15 | 0.93 | 0.77 | 0.93 | 1.15 |
| $L_9$ | 1.19 | 1.19 | 1.04 | 0.85 | 0.85 | 1.04 |
| $L_{10}$ | 1.15 | 1.23 | 1.15 | 0.93 | 0.77 | 0.93 |
| $L_{11}$ | 1.04 | 1.19 | 1.19 | 1.04 | 0.85 | 0.85 |
| $L_{12}$ | 0.93 | 1.15 | 1.23 | 1.15 | 0.93 | 0.77 |
| $L_{13}$ | 0.85 | 1.04 | 1.19 | 1.19 | 1.04 | 0.85 |
| $L_{14}$ | 0.32 | 1.19 | 1.6 | 1.68 | 1.6 | 1.19 |
| $L_{15}$ | 0.56 | 0.56 | 1.32 | 1.63 | 1.63 | 1.32 |
| $L_{16}$ | 1.19 | 0.32 | 1.19 | 1.6 | 1.68 | 1.6 |
| $L_{17}$ | 1.32 | 0.56 | 0.56 | 1.32 | 1.63 | 1.63 |
| $L_{18}$ | 1.6 | 1.19 | 0.32 | 1.19 | 1.6 | 1.68 |
| $L_{19}$ | 1.63 | 1.32 | 0.56 | 0.56 | 1.32 | 1.63 |
| $L_{20}$ | 1.68 | 1.6 | 1.19 | 0.32 | 1.19 | 1.6 |
| $L_{21}$ | 1.63 | 1.63 | 1.32 | 0.56 | 0.56 | 1.32 |
| $L_{22}$ | 1.6 | 1.68 | 1.6 | 1.19 | 0.32 | 1.19 |
| $L_{23}$ | 1.32 | 1.63 | 1.63 | 1.32 | 0.56 | 0.56 |
| $L_{24}$ | 1.19 | 1.6 | 1.68 | 1.6 | 1.19 | 0.32 |
| $L_{25}$ | 0.56 | 1.32 | 1.63 | 1.63 | 1.32 | 0.56 |

**Table S2**. Performance parameters of the utilized current sources in this work.

| Current sources | Current range | Accuracy |
|---|---|---|
| YOKOGAWA 2558A | 0.3 mA~60A | ±0.06% |
| Anmtake ATC70000 | 60A~300A | ±0.3% |
